# The mammalian target of rapamycin protein expression in human granulosa cell tumors

**DOI:** 10.4274/jtgga.galenos.2018.2018.0140

**Published:** 2019-11-28

**Authors:** Onur Güralp, Tugan Bese, Gamze Bildik, Fuat Demikiran, Ümit İnce, Eduard Malik, Macit Arvas, Özgür Öktem

**Affiliations:** 1 Department of Obstetrics and Gynecology, Carl von Ossietszky Oldenburg University, Klinikum AöR, Oldenburg, Germany; 2 Department of Obstetrics and Gynecology, İstanbul University Cerrahpaşa Faculty of Medicine, İstanbul, Turkey; 3 Koç University Graduate School of Health Sciences and School of Medicine, İstanbul, Turkey; 4 Department of Pathology, Acıbadem University Faculty of Medicine, İstanbul, Turkey

**Keywords:** Granulosa cell ovarian tumor, mTOR, rapamycin, ovarian cancer

## Abstract

**Objective:**

To investigate the role of mammalian target of rapamycin (mTOR) in human granulosa cell ovarian tumors and the therapeutic effect of rapamycin in COV434 mitotic granulosa cell lines.

**Material and Methods:**

A retrospective evaluation of the medical records and pathologic sections of patients with granulosa cell ovarian carcinoma was performed. mTOR and p-mTOR expression was immunohistochemically investigated. A *COV434* cell culture were treated with 0.5, 1, 2, and 5 μM rapamycin. Real-time growth curve analysis via xCELLigence system and apoptotic cell analysis via YO-PRO™-1 Iodide were performed to assess the therapeutic effect of rapamycin on cancer cells.

**Results:**

A total of twenty patients were evaluated. mTOR staining was detected in 18 (90%) patients. Mild, moderate, intense, and very intense staining was observed in three (15%), eight (40%), six (30%), and one (5%) sample, respectively. The mean mTOR staining ratio was 59±41%. P-mTOR staining was observed in two (10%) patients. One (5%) patient had 5% staining, and one (5%) patient had 100% staining for p-mTOR. Both of the latter patients had very intense staining. Rapamycin caused a dose-dependent growth arrest and induced apoptosis in *COV434* mitotic granulosa cells. The real-time growth curves of the cells treated with these drugs were distinguished by a marked reduced slope after exposure for several hours, indicating a rapid onset of apoptosis. Live/dead cell analysis with YO-PRO-1 staining showed that rapamycin induced apoptosis in 24% of the cells when used at 1 μM concentration, whereas the rate increased to 61% and 72% when the cells were treated with 2 μM and 5 μM rapamycin, respectively.

**Conclusion:**

mTOR expression is observed in various degrees in 90%, and p-mTOR expression is observed in only 10% of patients with granulosa cell ovarian carcinoma. Rapamycin caused a dose-dependent growth arrest and apoptosis in *COV434* mitotic granulosa cells.

## Introduction

Granulosa cell ovarian tumors constitute approximately 5% of all ovarian cancers. Contrary to the well-investigated epithelial ovarian tumors, there is very little known about the molecular and genetic changes in granulosa cell tumors. The studies are more or less focused on pathways playing a role in normal granulosa cell proliferation. The most important of these pathways is the follicle-stimulating hormone (FSH) pathway. Recently, a somatic missense mutation was revealed in the Forkhead box L2 (*FOXL2*) gene in 97% of adult-type granulosa cell tumors. Granulosa cell tumors are treated with surgery, adjuvant treatments (radiation or conventional chemotherapy), and hormone therapies (gonadotropin-releasing hormone antagonists, tamoxifen, and aromatase inhibitors). In metastatic or recurrent disease after primary surgical resection, adjuvant treatment is considered an option; however, the efficacy of systemic chemotherapy remains controversial. Based on our experiences on epithelial ovarian tumors, our first-line chemotherapy is platin-based chemotherapy. Agents such as doxorubicin, cyclophosphamide, vinblastin, bleomycin, and etoposide were combined with cisplatin, and the response rates were detected to be between 60-83% (1). With a better understanding of molecular and genetic features of granulosa cell ovarian tumors, treatment options will certainly increase.

mammalian target of rapamycin (mTOR) is a serine/threonine protein kinase and is part of the phosphatidyl inositole-3-kinase (PI3K)/AKT signal pathway. It plays a critical role in cellular development, metabolism, and the cell cycle of cancer cells (2).

Disturbances of the PI3K-dependent signalling pathway may lead to a variety of tumors including ovarian, endometrial, and cervical cancer. In clinical studies, first-line mTOR inhibitors were shown to have a promising clinical efficacy in ovarian and endometrial cancer (3). However, on a molecular level, the sensitivity or resistance rates of these agents are still unknown. At this point, we see that studies on the potential overactivity of mTOR pathway in granulosa cell ovarian tumors are scarce. In our study, we investigated the expression of mTOR and phospho-mTOR in granulosa cell ovarian tumor sections in order to better understand the molecular genetic features of granulosa cell ovarian tumors and determine whether mTOR inhibitors could be used in treatment. Moreover, we evaluated the possible therapeutic effects of rapamycin in COV434 mitotic granulosa cell lines.

## Material and Methods

The medical records and pathologic sections of patients who underwent surgery İstanbul University, Cerrahpaşa School of Medicine, Gynecologic Oncology Clinic between 1999 and 2011 and diagnosed as having granulosa cell ovarian tumor were evaluated retrospectively.

Our study was approved by İstanbul University, Cerrahpaşa School of Medicine, Medical Ethics Committee (Date: September 6^th^, 2011, No. 32476), and was supported by the İstanbul University, Scientific Research Fund of İstanbul University (Project number: 17384).

In our search of medical records and pathology archives, we detected paraffin sections of 20 patients with granulosa cell ovarian tumors.

### Pathological evaluation

Serial sections of 3 microns were cut from paraffin blocks using a Thermo Scientific^®^ microtome (MI, USA). The sections were incubated and dried at 80 °C for 20 minutes.

Immunohistochemical staining was performed using streptavidine biotine with a fully automated Ventana Benchmark Ultra (Arizona, USA) immunohistochemistry staining instrument. Deparaffinization of the sections was completed by incubation at 72 °C for 8 minutes followed by washing with Ez Prep (Ventana, USA). In the next step, sections were incubated with CC1 (Ventana, USA) at 95 °C for 8 minutes for the antigen retrieval process. Sections were incubated with primary antibody mTOR (7C10) Rabbit mAb (1:50, Cell Signalling Technology) at 24 °C for 48 minutes. Phospho-mTOR (Ser2448) (49F9) Rabbit mAb (IHC Specific) (Cell Signaling Technology, USA). Normal antibody Diluent (Scytek, USA) was used for antibody dilution. After treatment with primary antibodies, sections were treated with Blocker A and B (Ventana, USA) for 4 minutes each. Sections were incubated with biotinated secondary antibodies (IView DAB Delection Kit, Ventana, USA) and streptoavidine conjugated horseradish peroxidase (IView DAB Delecton Kit, Ventana, USA) for 8 minutes.

Sections were incubated with diaminobenzidine (DAB, IView DAB Delection Kit, Ventana, USA) and mordant application was performed by Copper (DAB, IView DAB Delection Kit, Ventana, USA). In all washing steps, Reaction Buffer (Ventana, USA) was used. Negative staining was performed using hematoxylin II (Ventana, USA) for 12 minutes. Sections were washed in tap water followed by alcohol baths. Xylene was used to clear the sections followed by covering with Consul-Mount (Thermo Scientific, UK) coverslip medium.

Mammarian ductal carcinoma in situ sections were used for positive mTOR. Normal mammarian gland sections were used for positive P-mTOR.

**Figure 1:**
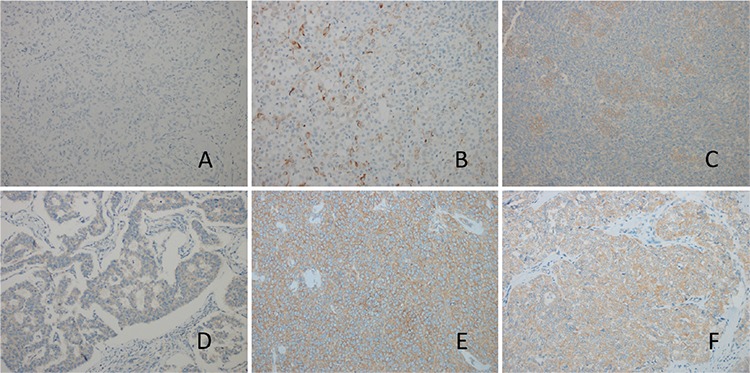
A-F. Scoring scales representation as percentage. mTOR and p-mTOR expression were evaluated semiquantitatively; the “staining extent” was defined as the percentage of staining. (A) No staining (×200); (B) 5% staining (×200); (C) 30% staining (×200); (D) 50% staining (×200); (E) 90% staining (×200); (F) 100% staining (×200) mTOR:Mammalian target of rapamycin

**Figure 2:**
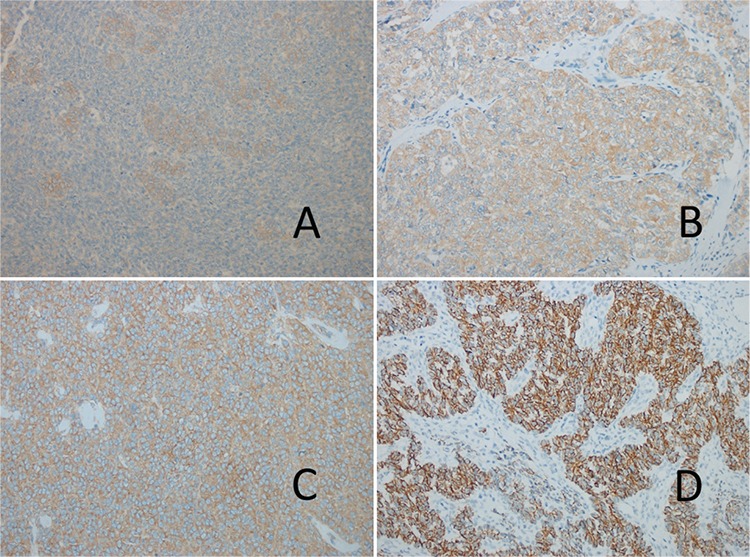
A-D. Scoring scales representation as intensity. The “staining intensity” was defined as “absent, weak, moderate, strong, and very strong”. (A) (+) staining; (B) (++) staining; (C) (+++) staining; (D) (++++) staining

Finally, sections were evaluated under a light microscope (Olympus BX 50, Olympus Corporation, Japan). M-TOR and P-mTOR expression were evaluated semiquantitatively; the staining extent was defined as the percentage of staining, and the staining intensity was defined as absent, weak, moderate, strong, and very strong. The scoring scales are represented in Figure 1A-F (percentage) and Figure 2A-D (intensity).

### Cell culture

A human immortalized granulosa cell line (COV434) was maintained in Dulbecco’s modified Eagle’s medium: F12 supplemented with 10% (v/v) FBS and 1% (v/v) penicillin-streptomycin *Amphotericin* B Solution (Gibco, 15240-062) at 37 °C with 5% CO_2_. The cells were routinely harvested using trypsinization with 0.25% trypsin–EDTA, and counted using a hemocytometer and 0.4% trypan blue.

### Real-time growth curve analysis via xCELLigence system

An xCELLigence System (ACEA Biosciences Inc. San Diego, CA, USA) was used according to manufacturer’s instructions. In brief, 100 μL of culture media was added to the each well, incubated at room temperature for 15 minutes, and the background impedance was measured. The trypsinized COV434 cells were centrifuged, resuspended in complete media, and seeded in a 96-well E-Plate at the density of 10.000 cells per well in a final volume of 200 μL. The cells were incubated at 37 °C with 5% CO_2_, and continuously monitored on the real-time cell analysis (RTCA) system at 30 minute intervals. When they reached the log growth phase, they were treated with 0.5, 1, 2, and 5 μM concentrations of rapamycine. The effects of rapamycin on viability and proliferation of COV434 cells were monitored on the RTCA system for up to 200 h. The results are expressed as normalized cell index (CI), which was derived from the ratio of CIs before and after the addition of the compounds. Recording and normalization of CI were performed using the RTCA Software 1.2.

### Apoptotic cell analysis via YO-PRO™-1 iodide

YO-PRO-1 is a carbocyanine nucleic acid stain used in identification apoptotic cells. Apoptotic cells become permeant to YO-PRO-1, whereas live cells are not stained with YO-PRO-1. Culture media of both the control and rapamycin-treated cells were aspirated and replaced with YO-PRO-1 containing culture media (1 μM). Hoechst 33342 was used as a counterstain. After 10 minutes of incubation at 37 °C with 5% CO_2_, they were observed under appropriate channels using an IF microscope (Olympus IX71, Japan).

### Statistical evaluation

Parametric variables are expressed as mean ± standard deviation, and non-parametric variables are expressed as median, minimum and maximum. Student’s t-test and analysis of variance were used for the comparison of parametric variables, and the chi-square test was used to compare nonparametric variables. Pearson’s correlation test was used for the evaluation of possible correlations between parametric variables, and Spearman’s correlation test was used for the evaluation of possible correlations between non-parametric variables. The Statistical Package for the Social Sciences (11.0, Chicago, IL, USA) was used for statistical evaluations. P<0.05 was accepted as significant.

## Results

A total of 20 patients with granulosa cell ovarian tumor were evaluated. The mean age was 46.05±11.5 (minimum: 26, maximum: 71). At the time of diagnosis, eleven (55%) patients were premenopausal and nine (45%) were post-menopausal. Eleven (55%) patients had stage 1a, five (25%) had stage 1c, one (5%) had stage 3b, and three (15%) had stage 3c disease. All patients had adult-type granulosa cell tumors. The mean tumor size was 92 mm ± 50 mm (minimum: 15 mm, maximum: 190 mm).

Necrosis was present in seven (35%) patients. The mitotic number was 1-4 in seven patients, 4-8 in eight patients, and more than 8 in three patients. Nuclear atypia was absent in one (5%) patient. Eight (40%) had mild, five (25%) had moderate, and two (10%) had severe atypia.

**Figure 3:**
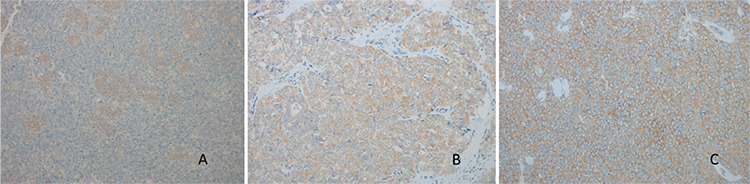
A-C. (A) 30% (+) mTOR staining granulosa cell ovarian tumor (×200); (B) 100% (++) mTOR staining granulosa cell ovarian tumor (×200); (C) 90% (+++) mTOR staining granulosa cell ovarian tumor (×200)

mTOR staining was not seen in two (10%) patients. Three (15%) patients had mild staining, eight (40%) patients had moderate, six (30%) had strong, and one (5%) patient had very strong staining. Mean mTOR staining percentage was 59±41 (minimum: 0, maximum: 100). mTOR statining features were given in Figure 3A-C.

There was no correlation between age and tumor size, mTOR staining percentage or intensity, p-mTOR staining percentage and staining. There was a positive correlation between mTOR staining percentage and staining intensity (p<0.001, r=0.819) (Table 1).

**Figure 4:**
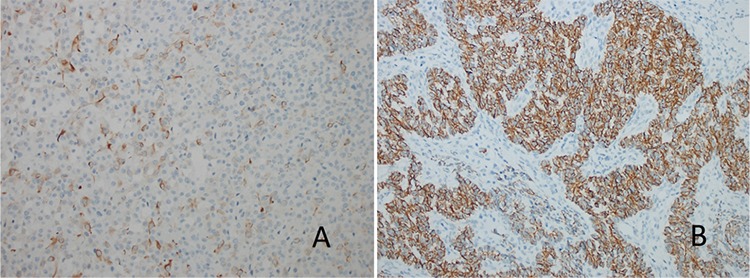
A, B. (A) 5% (++++) f-mTOR staining granulosa cell ovarian tumor (×200); (B) 100% (++++) mTOR staining granulosa cell ovarian tumor (×200)

P-mTOR staining was not observed in 18 (90%) patients. One patient had 5% and the other one had 100% staining. Staining intensity was very strong in both patients. P-mTOR staining features are presented in Figure 4A, B.

The patient with 5% p-mTOR staining was a 26-year-old woman with stage 3c disease, 5 cm tumor size, widespread tumor implants, 19 mitoses in 10 high-power field (HPF), and high-grade atypia. The patient with 100% p-mTOR staining was a 42-year-old woman with stage 1a disease, 12 cm tumor size, 2/10 HPF mitoses, and low-grade atypia. The low number of p-mTOR-positive cases rendered the statistical evaluation impossible.

**Figure 5:**
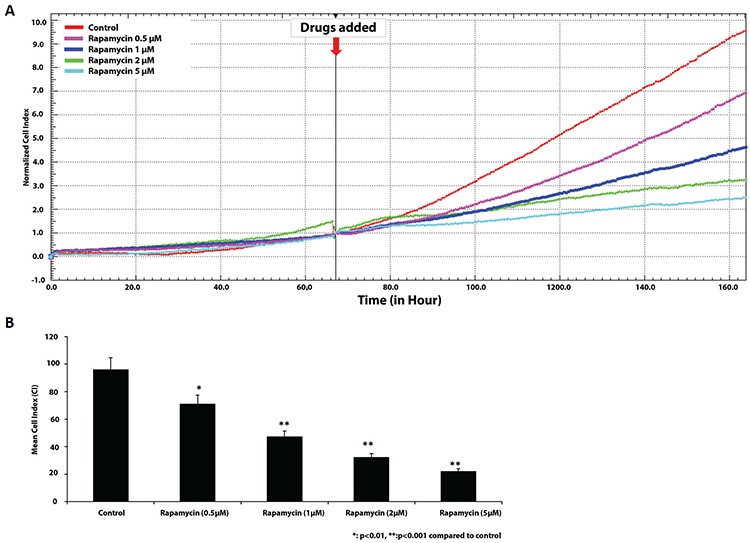
A, B. The real-time growth curves of the cells treated with these drugs were distinguished by a marked descendent curve after exposure for several hours, indicating a rapid decrease of the cell proliferation. (A) The real-time growth curves of the cells treated with rapamycin. (B) Mean cell index according to the various rapamycin doses

The growth curve characteristics of cells treated with different doses of rapamycin were analyzed to observe cell proliferation/apoptosis rate. Compared with the untreated control group, rapamycin caused dose-dependent growth arrest and triggered apoptosis in *COV434* mitotic granulosa cells. The real-time growth curves of the cells treated with these drugs were distinguished by a marked descendent curve after exposure for several hours, indicating a rapid onset of apoptosis (Figure 5A, B).

To further validate the findings obtained from the xCELLigence system and confirm apoptotic death after exposure to rapamycin, live/dead cell analysis with YO-PRO-1 staining was perfomed.

**Figure 6:**
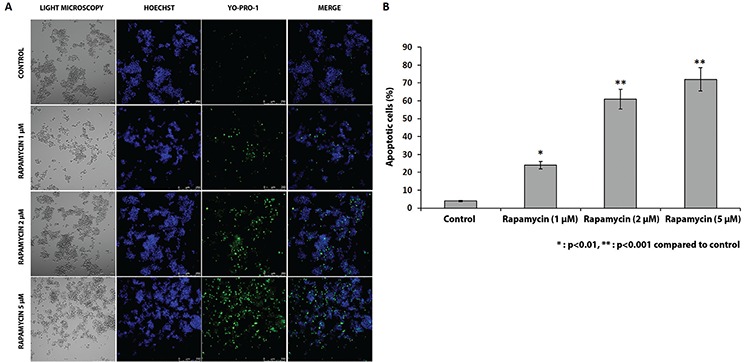
A, B. Live/dead cell analysis with YO-PRO-1 staining was carried out to confirm the apoptotic death after exposure to rapamycin. Overall, rapamycin induced apoptosis in 24% of the cells when used at 1 µM concentration, whereas the rate increased to 61% and 72% when the cells were treated with at 2 µM and 5 µM concentration, respectively. (A) Live/dead cell analysis with YO-PRO-1 staining (B) Rate of apoptotic cells according to the various rapamycin doses

Overall, rapamycin induced apoptosis in 24% of the cells when used at 1 μM concentration, whereas the rate increased to 61% and 72% when the cells were treated with at 2 μM and 5 μM concentration, respectively (Figure 6A, B).

## Discussion

mTOR is abundant in cytoplasm, especially in the perinuclear area in normal granulosa cells (4). The kinase active serine 24-48 phosphorylated form of mTOR, in other words the active form, p-mTOR, is generally increased during the M phase of the cell cycle. P-mTOR is observed near mitotic spindles and around contractile circle during cytokinesis. Inhibition of mTOR by rapamycin causes a dose-dependent decrease in granulosa cell proliferation and follicular development *in vitro*. However, in the presence of rapamycin, follicles do not undergo atresia in cell cultures. Yaba et al. (4) performed a study on rat ovaries and detected that mTOR expression was increased in cytoplasm compared with nuclei; they also showed that inhibition of mTOR in primary granulosa cell culture was associated with cell death in G2/M stages of cell cycle. Contrarily, granulosa cells survived in the presence of rapamycin, although tissue size was decreased. Therefore, rapamycin does not seem to stimulate folicle atresia directly, but it regulates follicular growth by acting as a check point.

Yu et al. (5) evaluated mTOR expression in mouse ovaries, primary mouse granulosa cells, and spontaneously immortalized rat granulosa cell lines (SIGC). mTOR expression was best seen in M phase in primary mouse granulosa cells and SIGC groups. In mouse granulosa cells, p-mTOR was detected to be increased in G2/M phase. In a recent study by Rico et al. (6), mTOR inhibition was shown to slow tumor development in a transgenic mouse model. In our study, mTOR staining was not seen in two (10%) patients, whereas three (15%) patients had mild, eight (40%) patients had moderate, six (30%) had strong, and one (5%) patient had very strong staining. P-mTOR staining was not observed in 18 (90%) patients. Detecting the stage of the cell cycle is possible in cell cultures but not in paraffinized sections. We cannot know for sure which stage of the cell cycle we observe when we stain sections for mTOR and p-mTOR. Therefore, this may be regarded as an inevitable confounding factor.

Although the role of mTOR and p-mTOR in the proliferation of granulosa cell and *in vitro* SIGC line is well-established, no rigorously validated immunohistochemical study or targeted therapy on human granulosa cell tumors has been reported to date.

The K-RAS oncogene is found in 48% of borderline and serous ovarian tumors (7,8). Stable transfection with H-RAS and other oncogenes may be used to immortalize granulosa cells (9). However, because RAS mutations have never been reported in ovarian granulosa cell tumors, the immortalized granulosa cell ovarian tumors may not represent the real human granulosa cell tumor proliferation mechanism. mTOR and p-mTOR are known to play a role in normal granulosa cell development and proliferation, but this pathway is not the only one that maintains protein expression. During cancer development, pathways other than mTOR could be activated.

Abnormal hyperstimulation of the pathways and oncogenic signalling are not the only pathologic mechanisms in cancer cell survival. Follicular growth and differentiation include complex mechanisms from the primordial stage until full establishment of the corpus luteum; less than 0.1% of follicles succeed. Female fertility depends on a delicate balance between survival signals for maturing follicle cells and death signals, leading the other to undergo atresia. For this reason, a disturbance in the apoptosis process may cause granulosa cell tumor development (1).

In cellular and molecular levels, ovarian cancer is known to be heterogenic. Altomare et al. (10) suggested that the PI3K pathway, which also includes mTOR, was active in 70% of all ovarian cancer types and its up-regulation was the main interfering factor in drug resistance. However, in this pathway, there are negative and positive feedback loops and alternative escape mechanisms interacting with the other pathways (11). For example, inhibition of mTORC1 by rapamycin leads to a short-term increase in mTORC2, which eventually increases the hyperstimulation of AKT. Hyperstimulation of AKT opposes the suppressive effect of mTOR inhibition. Besides, inhibition of mTORC1 leads to a loss of effective feedback of p70 and IRS-1 on each other. In addition to the PI3K and AKT pathways that are activated by FSH, there is an alternative MEK/ERK pathway activated by tyrosine kinases. There are cross-interactions between the above-mentioned pathways. Although normal granulosa cell proliferation depends on FSH signalling, proliferation may be accomplished without FSH in carcinoma cells.

In order to evaluate the mTOR pathway *in vitro*, the growth curve characteristics of cells treated with different doses of rapamycin were analyzed to observe mitosis/apoptosis rate. Compared with the untreated control group, rapamycin caused a dose-dependent growth arrest and apoptosis in COV434 mitotic granulosa cells. The real-time growth curves of the cells treated with these drugs were distinguished by a marked descendent curve after exposure for several hours, indicating a rapid onset of apoptosis.

To further validate these findings obtained using the xCELLigence system and confirm the apoptotic death after exposure to rapamycin, live/dead cell analysis with YO-PRO-1 staining was performed.

Overall, rapamycin induced apoptosis in 24% of cells when used at 1 μM concentration, whereas the rate increased to 61% and 72% when the cells were treated with at 2 μM and 5 μM concentration, respectively. These findings show that rapamycin may be a therapeutic option in vivo; however, further studies are needed to assess this hypothesis.

The main limitation of our study is the limited number of patients. Studies with a greater number of patients are needed to confirm the results of our study.

In conclusion, mTOR expression is observed in various degrees in 90% of patients with granulosa cell ovarian carcinoma, and p-mTOR expression is observed in only 10%. Rapamycin caused a dose-dependent growth arrest and induced apoptosis in COV434 mitotic granulosa cells, which was confirmed with live/dead cell analysis through YO-PRO-1 staining. There is a strong need for studies on expression of mTOR and p-mTOR in human ovarian granulosa cell tumor cultures.

## Figures and Tables

**Table 1:**
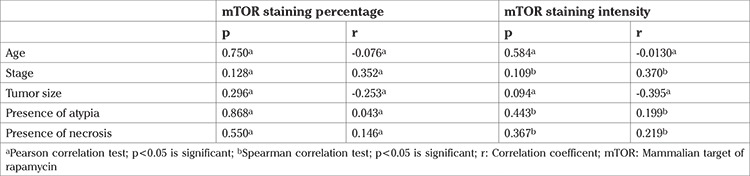
Correlations between age, stage, tumor size, presence of atypia and necrosis, and mTOR staining percentage and intensity
